# Spatiotemporal Clustering of Parking Lots at the City Level for Efficiently Sharing Occupancy Forecasting Models

**DOI:** 10.3390/s23115248

**Published:** 2023-05-31

**Authors:** Miratul Khusna Mufida, Abdessamad Ait El Cadi, Thierry Delot, Martin Trépanier, Dorsaf Zekri

**Affiliations:** 1 Laboratoire d’Automatique, de Mecanique et d’Informatique Industrielles et Humaines (LAMIH)-UMR CNRS 8201, Universite Polytechnique Hauts de France (UPHF) Mont Houy, 59313 Valenciennes, France; miratul.mufida@uphf.fr (M.K.M.); abdessamad.aitelcadi@uphf.fr (A.A.E.C.); dorsaf.zekri2@uphf.fr (D.Z.); 2 State Polytechnique of Batam, Batam 29461, Kepulauan Riau, Indonesia; 3 CIRRELT/Polytechnique Montréal, Department of Mathematics and Industrial Engineering, P.O. Box 6079, Station Centre-Ville, Montréal, QC H3C 3A7, Canada; mtrepanier@polymtl.ca; 4 ReDCAD Laboratory, University of Sfax, B.P. 1173, Sfax 3038, Tunisia

**Keywords:** parking occupancy forecasting, automated vehicle, parking profiles, spatiotemporal clustering, machine learning, model applicability

## Abstract

This study aims to address the challenge of developing accurate and efficient parking occupancy forecasting models at the city level for autonomous vehicles. Although deep learning techniques have been successfully employed to develop such models for individual parking lots, it is a resource-intensive process that requires significant amounts of time and data for each parking lot. To overcome this challenge, we propose a novel two-step clustering technique that groups parking lots based on their spatiotemporal patterns. By identifying the relevant spatial and temporal characteristics of each parking lot (parking profile) and grouping them accordingly, our approach allows for the development of accurate occupancy forecasting models for a set of parking lots, thereby reducing computational costs and improving model transferability. Our models were built and evaluated using real-time parking data. The obtained correlation rates of 86% for the spatial dimension, 96% for the temporal one, and 92% for both demonstrate the effectiveness of the proposed strategy in reducing model deployment costs while improving model applicability and transfer learning across parking lots.

## 1. Introduction

In the next 30 years, almost 70% of the world’s population is expected to reside in cities and adjacent areas [1]. These cities, therefore, have to be properly managed for supporting sustainable economic, social, and environmental development. A smart city is one that uses information and communication technology (ICT) to develop or improve core municipal infrastructures and services [2], such as transportation, public safety, and utilities [3]. Traffic management, mobility, and obviously parking assistance are certainly critical issues to handle in smart cities in order to reduce nuisances, such as traffic jams, pollution, time lost, safety, or stress caused by cruising for a free parking space in urban areas. For instance, finding vacant parking spots causes urban traffic congestion and generates between 5% and 10% of the traffic in the city, and even up to 60% in small streets [4]. Hence, the search for a vacant parking space results in unnecessary fuel consumption and pollution of the environment due to the emissions of gasses.

Parking occupancy data are, nowadays, increasingly available. For instance, the number of available spaces is widely shared to drivers of autonomous vehicles through digital boards in the city centers. Open data portals can be used to develop parking occupancy forecasting models. This allows designing solutions exploiting historical parking information to forecast the future occupancy of parking lots using machine learning (ML) or deep learning (DL) techniques [5,6,7,8,9,10]. The objective here is to help drivers of autonomous vehicles make better decisions concerning their travel planning [11] and guide them towards a parking lot close to their destination where they will actually find an available spot [12,13].

The research community has proposed effective solutions for forecasting parking occupancy using historical data. In the study by Mufida et al. [8], the authors have even proposed mechanisms for updating the models on the fly, which makes it possible to consider the deployment of parking assistance systems that can be used 24 h a day. Achieving good results with deep learning models requires determining optimal hyperparameters (e.g., optimizer type, number of layers, number of neurons per layer), which involves testing numerous combinations. A training phase with these parameters is then conducted before evaluating the model’s performance [14]. However, the current approach of tuning one forecasting model per parking lot has shown reasonable performance [3], resulting in significant effort when dealing with multiple parking areas in a medium or large city. Furthermore, developing a forecasting model is resource-intensive and time-consuming. Here, the cost refers to the time and resources required for deploying parking occupancy forecasting models.

However, different costs can be considered. For instance, the authors in [15] consider the cost for building an infrastructure for data collection. These costs encompass various aspects, including the establishment of infrastructure for data collection, as examined by Richter et al. [15]. In the context of this article, the term ’cost’ specifically refers to the combined expenditure of time and resources required for the successful deployment of parking occupancy forecasting models.

Our contribution in this article consists of designing an original framework that facilitates the sharing of parking occupancy forecasting models among multiple parking lots exhibiting similar spatiotemporal characteristics. Specifically, we propose a novel approach that not only maintains the quality of the forecasts but also achieves time savings by significantly reducing the number of forecasting models that need to be individually tuned for each parking lot. This can have a positive impact on the deployment cost of the models, which can be evaluated by considering several elements, such as the time spent on training, validation, and testing for each individual model, hyperparameters tuning execution time for each model with different hyperparameters combinations, model updating time, and the deployment time for clustering.

## 2. State of the Art

Numerous research works have focused on parking occupancy forecasting models. Some of them use classical models as regression models [16] and time series [17,18]. More recent studies exploit machine learning and deep learning models [8,19,20,21,22]. In this section, we first introduce machine-learning-based parking occupancy forecasting model. Then, we present several research works focusing on spatiotemporal correlations between parking lots.

### 2.1. Machine-Learning-Based Parking Occupancy Forecasting Models

Nowadays, machine learning (ML) and deep learning (DL) techniques are very popular and have led to the design of promising applications in the transportation domain [21,22]. Regarding concerns about parking occupancy forecasting, many solutions have been designed with various machine learning models both for parking lots [8,23,24] and in the context of on-street parking [25,26,27].

Various types of neural networks (NN) have been considered for designing parking occupancy forecasting systems. For instance, Shao et al. [27] aims to predict parking availability for different time frames in advance using a framework based on the recurrent neural network (RNN)/long short-term memory (LSTM) model. This model outperforms other state-of-the-art models, such as the static multi-layer perceptron (MLP). In [26], Yang et al. use graph convolutional neural networks (GCNN) to extract spatial features in large transportation networks incorporating multiple spatiotemporal data sources (traffic speed, weather, parking, etc.) and RNN/LSTM to capture the temporal features. Their work aims to design short-term parking occupancy prediction models using heterogeneous traffic data sources. The proposed system is effective in forecasting parking block occupancy 30 min in advance, especially for business and recreational areas. The use of RNN/LSTM is also considered in [8] to forecast parking lots occupancy at the city scale. In this work, the authors propose a mechanism to automatically determine the optimal hyperparameters for a neural network designed to forecast the future occupancy of a parking lot. Moreover, they consider the necessary updates to maintain the effectiveness of the forecast over time and thus propose a continuous forecasting service.

The solution proposed in [23] considers several machine-learning-based approaches to forecast the occupancy of parking lots, which in turn is used to determine occupancy-driven prices for arriving vehicles. In this work, several machine learning and statistical models, namely linear regression, decision trees, neural networks, and random forests, are evaluated. Forecasts of parking availability considering datasets of San Francisco, USA and Melbourne, Australia have been achieved in [28] using many different machine learning techniques, such as regression trees, neural networks, and support vector regression. Bayesian regularized neural networks have also been used in [10] to provide a reliable and fast forecast of available parking spaces. Camero et al. [6] highlight the challenges in developing single optimal model compared to the prevalent ones with numerous predictors. A new technique based on deep learning with recurrent neural networks is proposed to address the prediction of car park occupancy rate.

Prior studies address the development of an effective parking occupancy prediction model for each city parking lot. Yet, in a large and dynamic city, parking occupancy is influenced by various factors, such as population growth, economic development, urban planning, and seasonal changes, necessitating hundreds of models. This demands significant time investment for the hyperparameter tuning of machine-learning-based models.

### 2.2. Spatiotemporal Correlations between Parking Lots

Several approaches have been proposed to this point by the research community to characterize parking lots using spatial and/or temporal information to improve parking management. Richter et al. [15]. propose a method for predicting parking space availability using spatial and temporal clustering. The approach can help improve parking management by allowing users to plan their trips and reduce congestion caused by drivers searching for available parking spots. The study shows that the proposed method can significantly improve the accuracy of parking predictions compared to traditional methods. Wu et al. in [29] propose a clustering analysis method to identify the spatiotemporal patterns of on-street parking occupancy. The study shows that the method can help identify areas with high parking demand and optimize parking management strategies, such as adjusting parking fees and improving enforcement. The proposed approach can help reduce traffic congestion caused by drivers searching for parking spaces and improve the overall efficiency of parking management in urban areas. Bock et al. [30] propose temporal parking occupancy behavior based on citywide parking meter data. They compute an estimate of the parking occupancy of every parking lane equipped with a parking meter based on the validity period of the tickets sold. This occupancy information is averaged over all weekdays for specific times of the day. Moreover, it is possible to manage parking lots with both spatial and temporal properties, as in [26]. The paper [15] discusses the challenges associated with implementing such systems, including the high costs of deploying sensors and other infrastructure for data collection. The authors observe that the expense associated with the implementation of a parking forecast system may present a substantial challenge to its adoption. The installation of sensors and other infrastructure for data collection may be costly, as well as the development and maintenance of predictive models. When considering the cost of constructing parking occupancy forecasting models at the city level, one must take into account not only the creation and maintenance of individual models for single parking sites but also the time and resources necessary for implementing city-wide solutions [31].

In this work [26], Yang et al. do not characterize parking lots but use exogenous data related to temporal characteristics that change over time, such as weather, traffic, and parking transaction. These data are also considered as spatial characteristics since they depend on the parking location. In [28], Zheng et al. exploit data related to parking lots, which include the area name, the street name, the side of street, and street markers as spatial parking characteristics. Arrival and departure times are used as temporal parking features together with the duration of parking availability (in seconds). In [32], Ghosal et al. characterize parking lots with temporal properties, such as the time of the day, day of the week, holiday period, weather, and parking occupancy. They also consider the parking location as a spatial feature. The authors then present the clustering augmented learning method (CALM), which is embedded with deep learning models that combine CNN to extract spatial traffic flows and LSTM to learn temporal patterns to predict parking occupancy. In [33], Ionita et al. introduce the notion of parking demand pattern, which represents the parking behaviour in a urban area. They, therefore, label each parking area using where the parking is located to explain the parking occupancy evolution. The parking data considered in [33] include parking occupancy, traffic, weather, parking revenue, and the number of amenities.

The works discussed above show that there are limited studies that build a pattern to characterise a parking lot using both spatial and temporal features [15,26,28,29,32,33]. These works do not take into account several characteristics, such as exogenous factors, including the weather and the type of day (weekday or weekend), which have a significant impact on parking occupancy. The parking demand profile introduced in [33] is very interesting. However, it only relies on the parking occupancy usually recorded at peak hours or at periodic time intervals and the number of amenities from OpenStreetMap. Several data could not be integrated, such as the type of amenities, and exogenous factors, including weather information (temperature, precipitation, etc.) and the type of day (weekday, weekend).

Parking characteristics can be used in many studies related to parking lots, including measuring similarity to search for correlation between parking lots, which is the objective in our work. In this context, many machine learning models and techniques have been used so far to group parking lots. In [32], convolutional neural networks (CNN) are used to extract spatial linkages, whereas LSTM is used to capture temporal correlations. The authors also employ the clustering augmented learning method (CALM), which iterates between clustering and learning in order to create a robust learning process and categorize parking behavior in a city. In [32], Gomari et al. demonstrate that clustering parking event data can help to gain a better understanding of a city’s parking dynamics. The correlation between city parking temporal characteristics is also highlighted in [30]. In this work, Bock et al. define some similarity measurements using the mean-squared deviation of the average daily patterns in parking occupancy at the parking meters. Then, hierarchical clustering is applied based on this similarity measure to map hourly parking occupancy. The solution proposed by Ionita et al. [33] attempts to group parking spatial characteristics using unsupervised clustering with the K-means algorithm. The authors then investigate the exploitation of forecasting models developed on monitored on-street parking to unmonitored areas.

To summarize, the last two works [30,33] share the same objective as the framework we describe in this article, which consists of measuring parking lot similarity. However, the work in [30] focuses on the temporal parking characteristics and does not consider spatiotemporal correlations. In contrast to [30], Ionita et al. [33] consider both spatial and temporal characteristics to establish correlations between parking lots but with limited parking characteristics (parking occupancy, number of amenities) that can impact the quality of occupancy estimates for clusters. Furthermore, the authors do not consider the updates needed when new parking lots are considered or when changes occur in parking lot behaviour.

Therefore, to overcome the limitations noticed in [30,33], we propose a city-scale parking occupancy forecasting model using spatiotemporal clustering techniques based on parking characteristics to improve the accuracy of the parking occupancy forecasting model. We consider multiple spatial and temporal properties, such as parking lots nearby, number and type of relevant close amenities, exogenous factors (i.e., weather, day, time of the day, type of day (weekday or weekend), etc., to build a complete parking profile. The statistical analysis methods on the spatial and temporal attributes, such as those used in the the work of [7,8], enable the creation of a comprehensive parking profile. This profile allows understanding parking demand patterns, enhancing resource allocation, and facilitating informed decision making for parking management at different spatial and temporal levels. We implement correlation and principal component analysis (PCA) to select the best input features for further model deployments.

At the city level, if a new parking lot has to be integrated in our forecasting system or if changes occur regarding an existing parking lot affecting its occupancy trend [8], our framework can also be updated. Due to the dynamicity of parking occupancy that changes over time, the deployed parking occupancy forecasting model performance also tends to degrade over time [8]. Therefore, we need to maintain the model performance over time as new data become available. The updated mechanism will be explained in detail in Section 4.4.

## 3. Challenges and Methodology

**Figure 1:**
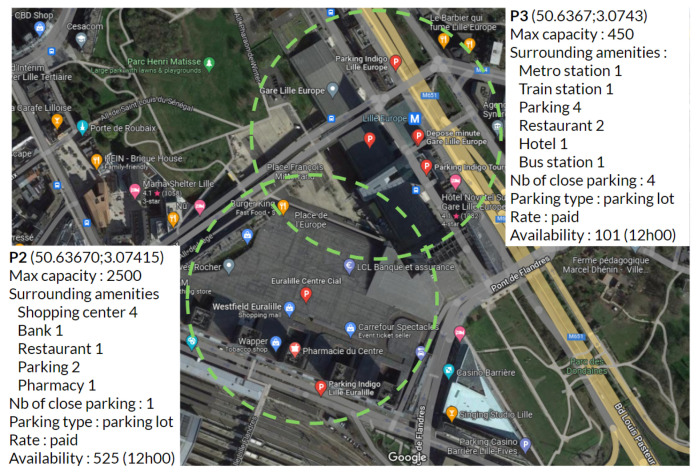
Illustration of parking lots characteristics showing parking area in the city of Lille, France. Adapted from Google Maps [34].

Developing a robust and reliable forecasting model that eases parking management is not an easy task, especially when one considers a real-world city-level deployment of the forecasting service. There are several important challenges to meet in this context. First, the different parking lots within the same city have their own spatial and temporal characteristics in terms of total capacity, occupancy trend, price seasonality, nearby facilities, etc., as depicted in Figure 1. The spatial character of parking refers to the location of parking spaces and how they are distributed within a given area, while the temporal character of parking refers to the number of occupied places, which obviously changes dynamically over time.

Regarding a city-level implementation of a parking occupancy forecasting system, the total cost for deploying forecasting models comes from the number of parking lots, the number of hyperparameters to tune the model, and the number of samples for training the forecasting models.

**Figure 2:**
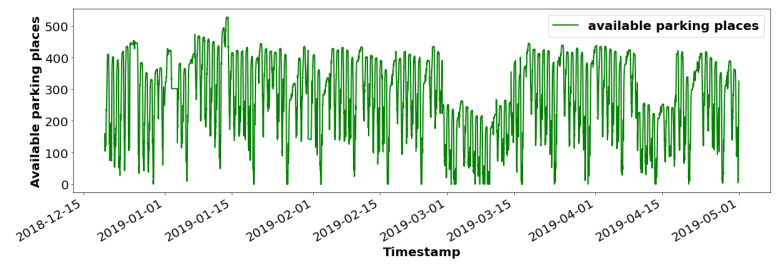
Parking dynamics change over time.

Additionally, the occupancy trend of a parking lot may sometimes change over time (See Figure 2) due to various factors (change in pricing or surrounding amenities, holiday period, road works nearby, sales in nearby stores, etc.). Hence, the efficiency of each forecasting model has to be regularly studied and updates have to be performed if necessary. Thereby, the cost to tune and deploy a forecasting model has not to be paid only once, at deployment time, but may occur several times. Hence, we study in the following the possibility to share a forecasting model between several parking lots. Therefore, we need to characterize each parking lot and establish a parking profile in order to determine whether a single forecasting model may be shared and provide good forecasts for different parking lots of the same city. This parking profile contains metadata about the parking and the facilities around it, which may influence parking occupancy.

This parking profile can be used to measure the similarity between the parking lots of the same city. Various similarity measurements can be applied here to identify the similarities, and clustering is the most common machine learning approach to group elements based on their similarities.

The challenge here is to explore how to obtain the optimal cluster implementation and then maintain the cluster consistency over time by entering into it only parking lots with the same behavior.

**Figure 3:**
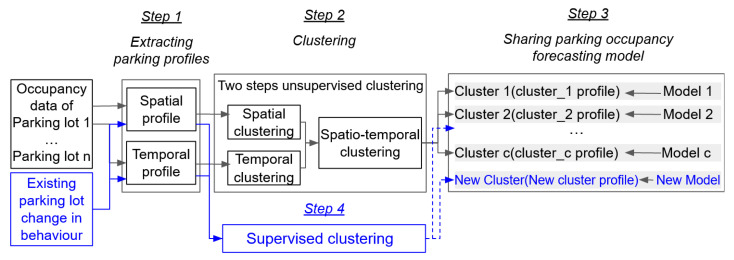
Workflow of our framework.

In this article, we propose an original framework to share parking occupancy forecasting models between several parking lots having similar characteristics. Our goal is to show that our approach both maintains the quality of the forecasts and enables saving time by significantly reducing the number of forecasting models to tune. The workflow presented in Figure 3 summarizes our approach and illustrates the methodology followed to design our framework. It consists of four main steps described in the following:

1. At the first step (detailed in Section 4.1), we define parking profiles for different parking lots in the city. A parking profile consists of spatial and temporal information characterizing a parking lot. It can be used to distinguish a parking lot from the others.
2. At the second step (detailed in Section 4.2), we perform a two-step unsupervised clustering method to group the parking lots according to their profiles. To do so, we develop several types of clusters, namely the spatial cluster, the temporal cluster, and, finally, the spatiotemporal cluster obtained by combining both previous ones.
3. At the third step (detailed in Section 4.3), based on these groups, we define the parking characteristic (parking profile) and the occupancy forecasting model for each cluster, and then we map applicability model and evaluate its performances.
4. At the fourth step (detailed in Section 4.4), we explain how we can update our framework if a new parking lot is considered or there is a change in the existing parking lot behaviour. In this case, we extract a new spatial or temporal profile and then perform supervised clustering, which can assign this new profile to the closest cluster or generate a new cluster.

## 4. Our Framework for Sharing Parking Occupancy Forecasting Models

In the following, we first introduce in Section 4.1 the concept of parking profile to characterise a parking lot. In Section 4.2, we propose a spatiotemporal clustering of parking lots at the city level.

In Section 4.3, we define for each cluster an occupancy forecasting model for the parking reference and propose a method to share this model with maintaining acceptable performances. We introduce a supervised clustering mechanism in Section 4.4 to enhance the models.

### 4.1. Parking Profile

The parking profile characterizes the parking behavior. It is determined by a variety of elements, such as the location, capacity, amenities (the number of different amenities around the parking and relevant amenity), weather, day, time of the day, type of the day (weekday or weekend), etc.

In our work, we consider the largest number of spatial and temporal properties to build a complete pattern as below:

- Spatial parking lot characteristics, defined by the spatial component, represent information describing where the parking lot is located, the maximum capacity, and information about the amenities surrounding the parking lot, especially their type (e.g., restaurants, railway stations, commercial centres, etc.) and number. The spatial profile thus tends to be less dynamic and will not change frequently. In addition, the maximum parking lot capacity is regarded as crucial information for spatial clustering. This information can be represented by the maximum capacity of the parking lots.
- Temporal parking lot characteristics are defined by dynamic information that changes over time and describes the parking lot occupancy trend. The parking dynamic also depends on exogenous factors, such as weather, time of the day, and day type.

Both spatial and temporal parking characteristics are important properties when defining an occupancy forecasting model, which may improve the forecast quality [3]. We thus integrate parking spatial and temporal features to define our parking profiles. In our work, the parking profile is defined as a tuple:

Parking_profile=(spatial_profile,temporal_profile)

The spatial part in the profile is defined as follows:

$$\begin{array}{c} Spatial\_profile=\{maximum\_capacity,geographical\_coordinates,amenities\_type,\\ relevant\_amenities,amenities\_number,parking\_nearby\} \end{array}$$

The temporal part in the profile is defined as follows:

$$Temporal\_profile={\left\{occupied\_places{\left(t\right)}_{i}\;,exogenous\_factors\left(t\right)\right\}}_{i\in [1,n]}$$

where

- $occupied\left(t\right)\_places_{i}$ is the number of occupied places in observed sequence *i* at time *t*.
- *i* is the frequency used for collecting the occupancy updates.
- *n* is the length of the sequence corresponding to the number of observations in the interval between occupiedplace(t) and occupiedplace(t+1). These observations are conducted in our case at each 5 min.

The temporal profile stores the number of occupied places, which changes dynamically over time. This attribute is a time series providing a set of observations collected with a constant frequency. In the temporal profile, exogenous factors represent the external aspects that potentially impact the parking lot’s occupancy over time. These exogenous factors integrate, for example, weather information (between 0 and 1) and the type of day, with a distinction between weekdays and weekends. The temporal profile is generated using one weekly parking occupancy window.

In the following, we explain how we exploit our parking profiles to identify similarities between parking lots of the same city and then share occupancy forecasting models.

### 4.2. Spatiotemporal Clustering of the Set of Parking Lots

Once defined, the parking profile can be used to find similarities between parking lots. We, therefore, provide a system to measure parking profiles’ similarity and group parking lots accordingly. Clustering is an essential data mining technique for grouping data points into homogeneous groups (or clusters). Clustering techniques are well-known to provide a simple solution for spatial and temporal grouping [3,5,15,29,35,36]. In our work, we try to group similar spatial and temporal parking lot profiles. The parking lots are provided without prior knowledge regarding their membership in a group. Therefore, our spatiotemporal clustering solution exploits an unsupervised approach to discover groups of parking lots sharing the same occupancy trends.

In order to group the parking lots according to their profiles, we apply a two-step unsupervised clustering process. We thus build several types of clusters, namely the spatial cluster and the temporal cluster to finally obtain the spatiotemporal cluster obtained by combining both previous ones.

More precisely, we exploit for the spatial dimension a straightforward yet successful method based on K-means clustering with Euclidean distance (ED) [37], and dynamic time warping (DTW) [38] for the temporal one. K-means provides a fair trade-off between the quality of the solution found and the computational cost [39]. K-means has several benefits compared to other clustering algorithms since it is suitable for large unlabeled datasets and has a linear time complexity with large datasets.

#### 4.2.1. Spatial Cluster

In this section, we focus on spatial clustering. In order to group parking lots according to their spatial profile, we consider two distinct types of input in our clustering process:

1. Numerical inputs, such as *maximum capacity*, *longitude*, *latitude*, *number of parking nearby*, and the *number of amenities per type* around (amenity distribution), can be clustered in the straightforward mechanism for numeric.
2. Categorical inputs, such as relevant amenities, require mixed input type clustering techniques, such as K-prototype [40].

To compute our spatial cluster, we use the parking spatial profile as an input vector. Initially, we place the cluster centroid randomly. The centroid is then relocated based on the computed average distance of each member (spatial profile) of the spatial cluster to its centroid using Euclidean distance. This calculation is repeated until the process converges and there are no more cluster assignments.

Before applying clustering algorithms to the spatial profile, numerical data are normalized. In the case of longitude and latitude, normalization is typically not necessary. This is because longitude and latitude are already in a standard range to avoid bias and to use a consistent input scale. We thus target values in the 0 to 1 range that are computed with Equation (1).

(1) $$X_{normalized}=\frac{X-X_{Min}}{X_{Max}-X_{Min}}$$

where

- $X_{Min}$ is the smallest value in the dataset before normalization.
- $X_{Max}$ is the largest value in the dataset before normalization.
- $X_{normalized}$ is the value of the data point after normalization.
- *X* is the original value of the data point before normalization.

Based on the spatial profile, we generate spatial dissimilarity matrix using the Euclidean distance, computing six main elements as an input, calculated using Equation (2).

(2) $$ED_{ij}^{2}=\sqrt{\sum_{Att=1}^{n}{\left(X_{Atti}-Y_{Attj}\right)}^{2}}$$

where

- $ED_{ij}$ is Euclidean distance
- Att is attribute
- $X_{Atti}$ is the start point of attribute *i*
- $Y_{Attj}$ is the end point of attribute *j*

It compares the pairwise distance of each spatial profile. A chosen measure of distinction between the *spatial profile*(*i*)th and *spatial profile*(*j*)th object is equal to the value of the (*ij*)th element in this square-symmetrical spatial profile matrix. The diagonal elements are equal to zero. Then, we group the spatial profiles using K-prototype approach [40], that is, by combining K-means for numerical with K-modes for categorical.

We need to preprocess the data by converting categorical variables (relevant amenities in our case) into numerical variables. One common method is to use one-hot encoding, where we create binary columns for each category and assign a value of 1 to the corresponding column for each data point. Then, we apply K-means clustering to the numerical data to group the similar numerical data points together. We choose the optimal number of clusters using silhouette score. Afterward, we apply K-modes clustering to the one-hot-encoded categorical data to group the similar categorical data points together. We again choose the optimal number of clusters using silhouette score. Once we have clustered both numerical and categorical data, we can combine the clusters by assigning each data point to the nearest numerical cluster and nearest categorical cluster. We use the Euclidean distance matrix to measure the similarity between data points. Finally, we evaluate the results of the combined K-means and K-modes clustering by calculating the silhouette score, which measures the similarity of a data point to its own cluster compared to other clusters. A higher silhouette score indicates better clustering performance.

#### 4.2.2. Temporal Cluster

The temporal clustering groups the parking occupancy patterns according to trend, seasonality, and cycle, which change dynamically over time. For the clustering task, we use K-means to temporally group the parking areas based on their profiles. There are two well-known matrices to measure the distance or similarity between two series, which are Euclidean distance (ED) [37] and dynamic time warping (DTW) [38]. The limitation with the use of Euclidean matrices for time series clustering resides in the fact that Euclidean distance requires series of same length. When there are temporal shifts, the correlation between the two series is not correctly determined. Hence, we apply DTW for temporal distance measurement and grouping in our approach to obtain better temporal clusters.

The DTW distance between two time series $X=(x_{1},x_{2},\ldots ,x_{n})$ and $Y=(y_{1},y_{2},\ldots ,y_{m})$ is obtained using Equation (3):

(3) $$D_{DTW}\left(X,Y\right)=D_{base}\left(x_{1},y_{1}\right)+min=\left\{\begin{array}{c} D_{DTW}\left(X,Y\left[1:-\right]\right)\\ D_{DTW}\left(X\left[1:-\right],Y\right)\\ D_{DTW}\left(X\left[1:-\right],Y\left[1:-\right]\right) \end{array}\right.$$

where $D_{base}\left(x_{1},y_{1}\right)$ is the base distance: $D_{base}\left(x_{i},y_{j}\right)=\left|x_{i}-y_{j}\right|$.

Dynamic time warping (DTW) is a technique used to compare two time series sequences, even if they have different lengths, by finding the optimal alignment between them.

In DTW, a cost matrix is computed between the two sequences to represent the pairwise distance between each element in the two sequences. The cost matrix is then used to find the optimal warping path, which is the path through the matrix with the lowest total cost. To compute the cost matrix efficiently, many implementations use a binary matrix representation and corresponding elements (this refers to the element in one sequence that is matched or aligned with a specific element in the other series based on the DTW algorithm). This is because matching elements are typically small and can be processed quickly, making them well-suited for computing the cost matrix efficiently.

In the binary matrix representation, each element in the matrix is either 0 or 1, depending on whether the two corresponding elements in the time series match or not. This representation reduces the dimensionality of the problem and allows for efficient computation of the cost matrix.

#### 4.2.3. Spatiotemporal Cluster

Considering the spatial and temporal cluster deployment, we define a spatiotemporal cluster by combining both of them using the Cartesian Product operator:

$$\left\{Spatial\_Cluster\right\}X\left\{Temporal\_Cluster\right\}=\{\left(Spatial\_Cluster_{i},Temporal\_Cluster_{j}\right)\}$$

with

- $Spatial\_Cluster_{i}\in \left\{Spatial\_Cluster\right\}$
- $Temporal\_Cluster_{j}\in \left\{Temporal\_Cluster\right\}$

We design spatial and temporal clusters separately because the characteristics of the parking spatial profiles are less dynamic than those of the temporal profiles where parking occupancy evolves over time.

We combine the two clustering approaches to obtain a multi-clustering result. Indeed, as spatial and temporal features are not suitable for being handled together, our multi-clustering approach helps to define a cascade of clusters. In this way, we could organize the parking lots into meaningful groups from different perspectives.

#### 4.2.4. Cluster Evaluation

A common limitation of K-means implementation resides in identifying the best *k* or the number of clusters. The elbow approach [41] and silhouette analysis [42] are two popular visual methods for determining the ideal number of clusters. Both these methods are used in our study to guarantee the clustering quality and identify the target number of spatial and temporal clusters for the set of parking lots.

The quality of our clustering is computed using the silhouette score using Equation (4). When applied in comparison with all other clusters, this index evaluates how similar to its own cluster each individual observation is.

(4) $$SilhouetteScore=\frac{\left(b-a\right)}{max(a,b)}$$

where

*a* is the average distance within each item in the cluster

*b* is the average distance between the clusters

The silhouette score is a metric used to evaluate the quality of a clustering algorithm’s output. It measures how similar an object is to its own cluster compared to other clusters. A silhouette score ranges between −1 and 1, where a score closer to 1 indicates a well-clustered data point, while a score closer to −1 indicates that the data point may belong to the wrong cluster.

### 4.3. Sharing Parking Occupancy Forecasting Model

Once the spatiotemporal clusters are defined, our goal is to exploit them to facilitate the deployment of a parking occupancy forecasting model at the city scale. Our goal here is to share parking occupancy forecasting models among several parking areas (located in the same cluster) to avoid paying the high tuning cost when deploying one model per parking lot independently. Obviously, when sharing models between several parking areas, we have to preserve a good forecast accuracy. In the following, we explain how we create a reference forecast model developed for a single parking lot and adapt this model for the other parking lots belonging to the same cluster.

Initially, we define a reference parking lot. Its profile is selected by computing the closest parking profile to the cluster centroid by obtaining the smallest distance between centroid and parking lots in each cluster. Related to this reference profile, based on our previous work [8], the reference parking occupancy forecasting model (i.e., RNN-LSTM) is tuned and trained. Its quality is evaluated using MAPE, calculated using Equation (5).

(5) $$MAPE=\frac{1}{n}\sum_{t=1}^{n}\left|\frac{X_{t}-{\hat{X}}_{t}}{X_{t}}\right|$$

A reference model is a model chosen from the cluster and developed to represent the parking profile at the same cluster. It can be used to create forecasts without any further adjustments because reference models are typically simple and quick to implement.

We have a list of:

- the dissimilarity between parking reference to the other parking lot in the same clusters and different clusters.
- the model performance of forecasting model that is trained and tested in the same cluster.

Afterward, we compute the correlation between the list of dissimilarity and the list of model performance that represent parking lots in the same cluster using Equations (6) and (7). We iterate the same steps for parking lots that belong to different clusters. To share the forecasting model, we examine the correlation between the dissimilarity amongst parking lots and the forecasting model performance.

To know the measure of the linear relationship between continuous features (model performance and the distance) amongst parking lots, we use Pearson correlation. The Pearson correlation coefficient assesses the statistical link, or association, between two continuous variables. It provides information on the amount and direction of the relationship’s link, or correlation. Equation (6) is used to compute the Pearson correlation coefficient (*r*) between two random variables, *x* and *y*.

While coefficient determination or $R^{2}$, in Equation (7), expresses the fraction of the variance in dependent variables caused by independent factors, *Y* represents the dependent variable’s actual value, $\bar{Y}$ is dependent variable’s mean value, and $\hat{Y}$ represents prediction value [43].

(6) $$r=\frac{\sum \left(x-\bar{x}\right)\left(y-\bar{y}\right)}{\sqrt{\sum (x-\bar{x}{)}^{2}\sum (y-\bar{y}}{)}^{2}}$$

(7) $$R^{2}=\frac{\sum (Y-\hat{Y}{)}^{2}}{\sum (Y-\bar{Y}{)}^{2}}$$

To recapitulate, one of our contributions in this article consists of designing an original framework that facilitates sharing of parking occupancy forecasting models among multiple parking lots exhibiting similar spatiotemporal characteristics. Specifically, we propose a novel approach that not only maintains the quality of the forecasts but also achieves time savings by significantly reducing the number of forecasting models that need to be individually tuned and trained for each parking lot. This obviously has a positive impact on the deployment cost of the models by reducing several items, such as the time spent on offline training, validation, and testing for each individual model, hyperparameters tuning execution time for each model with different hyperparameter combinations, model updating time, and the deployment time for clustering.

It is also important to mention that the cost needed to deploy a city-level parking occupancy forecasting model will vary significantly depending on the number of parking lots to consider, the number of hyperparameters to tune, and their possible combinations (the size of hyperparameter search space), the number of samples per hyperparameter considered for training, validation, and testing. A thorough assessment of these factors is necessary to estimate the precise cost associated with the model deployment. An experimental cost analysis is detailed in Section 5.4.

Concerning the hyperparameters tuning, the search space size depends on the combinations of several elements, such as the learning rate, number of layers, number of neurons per layer, the optimizers, and activation function. Table 1 details the search space of hyperparameters tuning. The elements presented in this table are used to generate combinations that we used to run our experiments in order to find the optimal forecasting model.

Hence, there exist approximately 270 million potential combinations within the provided hyperparameter tuning search space. It is challenging to estimate the overall execution time for training the 270 million possible combinations in the provided hyperparameter tuning search space. Several factors, including hardware performance, software implementation, dataset size, and computational complexity, all contribute to the difficulty. We can, however, make a preliminary estimate based on certain assumptions. We may compute the overall execution time by assuming an average training time of one hour for each model configuration:

TotalExecutionTime=270,950,400×1h≈3000years

Based on this number, using grid search, which tests every possible combination, it appears to be impossible to achieve, which is why we opted for the random search method with randomly testing 100 combinations from the search space, which takes approximately 4 days to train a model. However, if we apply the same mechanism to create a model for each parking lot at the city level, the cost would increase significantly. By using our clustering and shared model approach, we can significantly reduce the number of models that need to be trained. This reduction in models leads to a substantial decrease in the time needed for deploying forecasting models.

It is crucial to note that this estimation neglects to account for any overhead time, data loading, preprocessing, or computer environment constraints, such as session duration constraints.

Furthermore, the actual execution time may vary based on a variety of parameters, including the complexity of the RNN-LSTM model, dataset size, and implementation efficiency. Using these estimations, the total execution time for computing the whole search space is anticipated to be in the billions of hours. The lengthy execution time may not be feasible within the chosen computer environment. Thus, it is recommended to explore alternative computing resources, such as cloud-based platforms equipped with high-performance GPUs or distributed computing systems, to effectively handle the extensive processing workload. These options offer the required computational power and scalability for efficient exploration of the vast hyperparameter tuning search space. However, it is important to consider the associated financial costs of providing a suitable machine and environment for hyperparameter tuning.

### 4.4. Supervised Clustering for Updating Models

**Figure 4:**
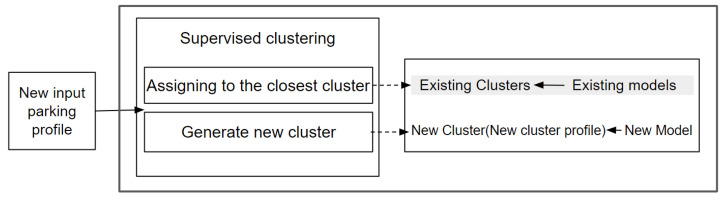
Workflow for updating mechanism.

At the city level, if a new parking lot has to be integrated in our forecasting system or if changes occur on an existing parking lot affecting its occupancy trend, our cluster framework has to be updated. Therefore, our framework has to determine if an existing model can be applied to the new or updated parking lot. Figure 4 illustrates the workflow of the model update process.

Our framework exploits the spatial and temporal profiles using the pattern explained in Section 4.1.

- At the first step, using the new profiles, our system performs supervised clustering by computing the distance between the new parking profile and the reference parking profiles of each cluster.
- At the second step, we assign this new profile to the closest cluster; this means that it can reuse an existing model for existing clusters that were formed previously. However, there is a possibility that the new parking lot generates a new profile and a new cluster and therefore a new model because the new profile is not similar to any existing profile, so it is not classified in any existing cluster. The new cluster generation happens due to the fact that the new input parking profile is not similar to any of the existing cluster profiles. Involving this new parking profile in existing clusters will cause cluster quality deterioration. Thus, generating new clusters is an effort to maintain cluster quality as new parking profiles emerge. Thus, we can maintain the (clustered) model performance over time.

## 5. Computational Experiments

In this section, we discuss the experimental results we obtained to show the effectiveness of our framework.

### 5.1. Dataset and Library

**Figure 5:**
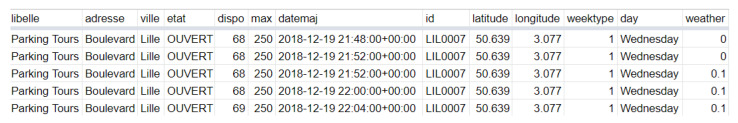
Parking data input.

Our models were built and evaluated using real-time parking data provided by the Métropole European Lille (MEL), the largest metropolis in the north of France, with a population of 1.2 million people. The dataset comprises information on the occupancy of 17 parking lots that contained more than 15,000 parking spaces for a period ranging from December 2018 to April 2019. The number of parked vehicles is updated every 5 min on average, and the information is freely accessible (https://opendata.lillemetropole.fr/explore/dataset/disponibilite-parkings/information/ (accessed on 12 February 2020)). The raw data were saved in csv files every day, as shown in Figure 5. Each tuple in the files has 12 attributes: label (parking name), address, city, status (open or closed), number of available spots, maximum capacity, date, parking id, coordinates, geometry, display panels, timestamp, and parking id.

In the raw data used to deploy the model, values are sometimes missing. We therefore initially undertake a data cleaning phase to ensure that the dataset is compatible with the training stage. As a consequence, missing values are filled by interpolating data according to the time and sequence. Other approaches could be used to handle missing data, such as the bagging learning approach proposed in [44]. For example, if values are missing on Monday 08.00–11.00, we assume that the parking occupancy is the same as the previous Monday to replace null values.

In the following, we discuss the results obtained for the experiments carried out using several machine learning frameworks, namely *tslearn*, *tensorflow*, *dtw*, *dtaidistance*, and *keras*.

### 5.2. Spatiotemporal Clustering

The parking profile consists of the spatial and temporal profiles as explained in Section 4.1. An example of a parking profile is provided in Table 2. Let us note that the information related to amenities is collected using the overpass turbo API on each parking lot’s location using a radius of 500 m around.

**Figure 6:**
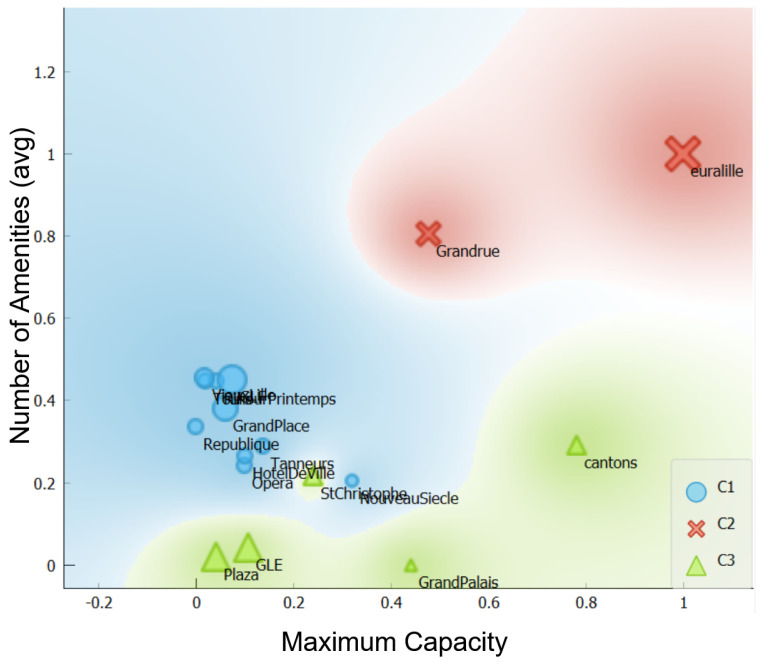
Example of spatial clusters generation.

The cluster deployment, as described in Section 4.2, begins with the spatial and temporal clustering, which are combined to generate spatiotemporal clusters. For the spatial clustering, we use a combination of K-means for numerical elements and K-modes for the categorical ones. These two algorithms use the six inputs (maximum capacity, longitude, latitude, parking nearby, relevant amenity, and amenity type) as mentioned in Table 2. We start by generating the spatial matrices dissimilarity using Euclidean distance, computing the elements of the spatial profile as an input, and then we group the spatial profiles using K-prototype (K-means and K-modes), as explained in Section 4.2.1. As an example, we present in Figure 6 an intermediary result of the spatial cluster output, which represents maximum capacity over amenity types (number) distribution.

Table 3 represents the output of our spatial clustering, which has three clusters. Each line illustrates the parking lot clusters profile related to the average of all parking lots in each cluster, the maximum capacity, average parking nearby, list of relevant amenities, and the amenity types (number).

**Figure 7:**
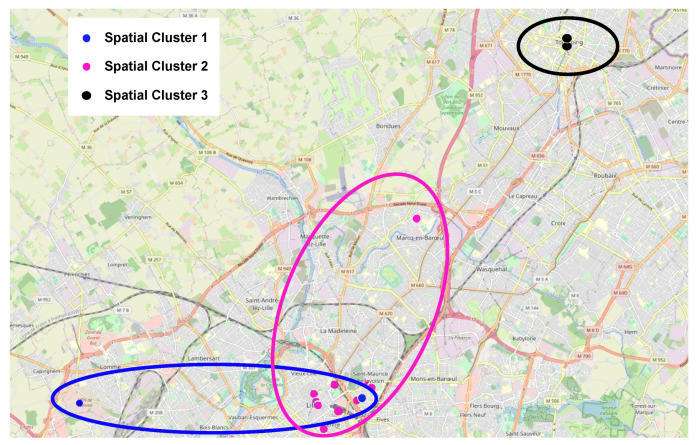
Three spatial clusters.

The spatial clustering result is shown in Figure 7. We can observe that three clusters are generated for the parking lots of our dataset. There is a cluster overlap in our spatial cluster because we are using six different inputs, not only the parking lot location.

For temporal clustering, we apply K-means using DTW as a distance matrix. To generate our temporal clusters, we used two features (parking occupancy and exogenous factors (weather, type of the day)) as inputs, as presented in Table 2. For each time series in the dataset, we need to calculate the DTW distance to each of the K cluster centers. Then, we assign the time series to the cluster with the nearest center. Next, the cluster centers update, and, once all data points have been assigned to clusters, update the center of each cluster as the mean of all the time series assigned to that cluster. We repeat those procedures until the centroid converges.

**Figure 8:**
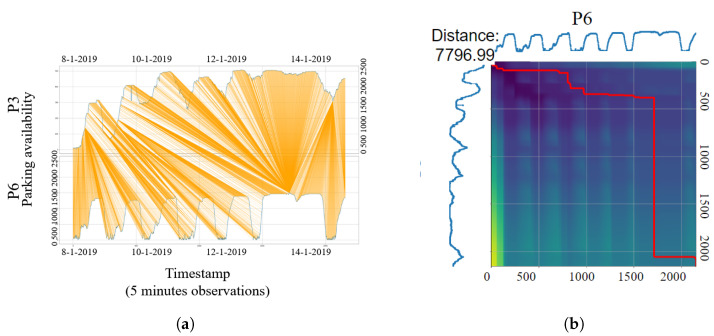
Temporal distance (DTW) path between P3 and P6 in binary matrix representation and P3 matching element to P6. (**a**) The DTW matching element between P3 and P6. (**b**) Binary matrix representation of DTW path between P3 and P6 (The red line represents the shortest path between two series computed by DTW).

**Figure 9:**
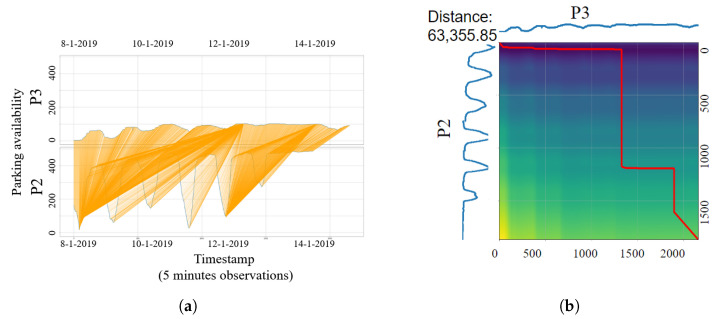
Temporal distance (DTW) path between P2 and P3 in binary matrix representation and P2 matching element to P3. (**a**) The DTW matching element between P2 and P3. (**b**) Binary matrix representation of DTW path between P2 and P3 (The red line represents the shortest path between two series computed by DTW).

To explain more about how dynamic time warping (DTW) works, we depict three parking lots (P2, P3, and P6) in our dataset to demonstrate the computational processes of K-means temporal clustering. Figure 8 and Figure 9 propose two examples of dissimilarity measurement between three parking occupancy series using DTW. DTW can be represented as matching elements of two parking occupancy time series, as shown in Figure 8a and Figure 9a, or as binary matrix, illustrated in Figure 8b and Figure 9b (a detailed explanation is presented in Section 4.2.2). Matching elements illustrates the pairwise distance between two series (represented in Figure 8a for P3 and P6 and Figure 9a for P3 and P2). However, a binary matrix is a cost matrix to calculate the minimum distance between two time series (Figure 8b and Figure 9b).

Figure 8 demonstrates that P3 and P6 have a smaller dissimilarity compared to P3 and P2 in Figure 9. Indeed, the DTW calculation shows that P6 is closer to the centroid P3, with a temporal distance equal to 7796.99 between the two parking lots shown in Figure 8b, which is smaller than the average cluster distance calculated by K-means, which means that P3 and P6 belong to the same temporal cluster. However, the temporal distance between P3 and P2, equal to 63,355.85 and shown in Figure 9b, is larger than the average cluster distance calculated by K-means for the first cluster where P3 is a centroid, which explains that P3 and P2 belong to different temporal clusters.

**Figure 10:**
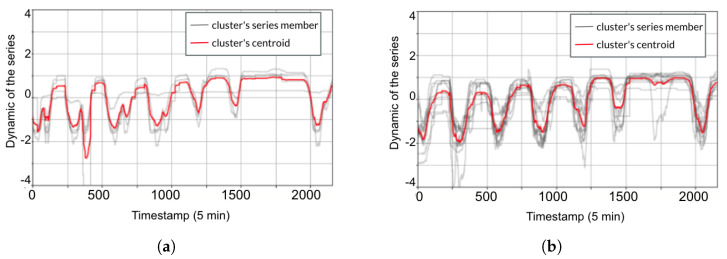
K—means clustering with dynamic time warping (DTW). (**a**) Temporal cluster 1. (**b**) Temporal cluster 2.

In conclusion, the temporal cluster development observed in the three parking lots can be generalized to all the parking lots in the same city by applying the same process of the DTW between each parking lot to the cluster centroids to generate a dissimilarity matrix. By doing so, we group parking lots in the city based on their temporal characteristics similarity to identify a temporal cluster, as presented in Table 4 and illustrated in Figure 10. Each temporal cluster contains parking lots that have similar temporal profiles.

**Figure 11:**
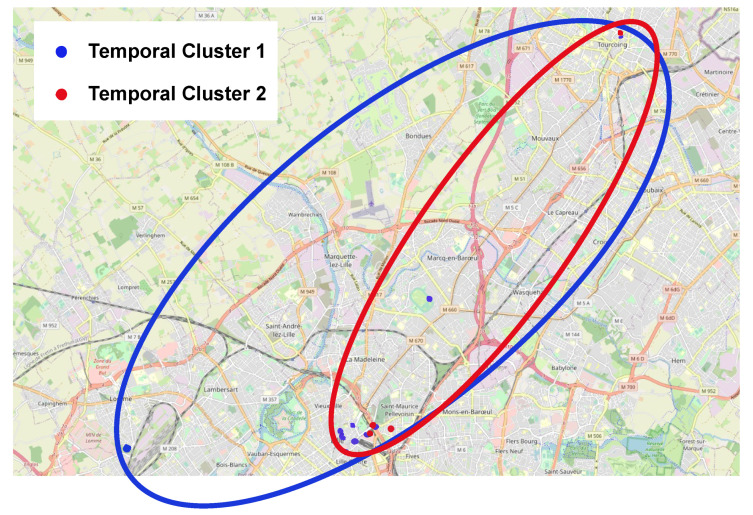
Two temporal clusters.

The results of the temporal clustering applied to our dataset projected in the geographical representation are depicted in Figure 11. The temporal cluster represents parking lots that have similar temporal behaviors. Figure 11 also illustrates that parking lots that are located close geographically do not always have the same evolution over time. Thus, they are clustered differently.

Once the clusters are generated, we evaluate their quality using the silhouette score using Equation (4). The higher the silhouette score, the better it is. The best cluster is the one providing the shortest distance within the elements belonging to the cluster and the greatest distance between the other clusters.

In order to determine whether the clustering process delivers the appropriate number of clusters, we use the elbow approach and silhouette analysis [45]. The elbow technique with the greatest silhouette score provides the optimal *k*. We obtained optimal silhouette scores equal to 0.549 for the three spatial clusters and equal to 0.791 for the two temporal clusters.

To generate spatiotemporal clusters, we combine the spatial cluster in Table 3 and temporal cluster in Table 4 using the Cartesian Product operator as explained in Section 4.2.3. The five resulting spatiotemporal clusters are presented in Table 5. We can cite as an example one spatiotemporal cluster made up of two parking lots, P1 and P16, obtained with the combination of temporal cluster 1, made up of parking lots P3, P6, P1, and P16, and spatial cluster 1, with parking lots P1, P9, P10, P11, P14, and P16.

### 5.3. Sharing Parking Occupancy Forecasting Models

Once the spatiotemporal clustering is performed, we aim to share models between parking lots in the city using the clustering results obtained in the previous steps. Using clustering information as a distance matrix, we demonstrate the possibility to share parking occupancy models amongst parking lots in the city. In order to do so, we try to measure correlation between spatiotemporal distances and parking occupancy models performance for parking reference to the other parking lots, as mentioned in detail in Section 4.3.

We distinguished our observation, namely spatial clusters, temporal clusters, and spatiotemporal clusters, to know which clusters have higher correlation. Afterward, we depict P3 as parking reference and train and tune a parking occupancy model for P3. We compute the DTW distance between parking references to other parking lots. We test the trained model of parking reference P3 using another testing set from another parking lot to evaluate the model sharing feasibility.

Table 6 demonstrates an example of the correlation between spatial parking dissimilarity and parking model performance. P3 is a parking reference for spatial cluster 1; P6 and P12 are in the same spatial cluster as P3. The spatial dissimilarity between P3, P6, and P12 is very small. Thus, P6 and P12 can share the model (which is trained and optimized) with P3 since their test results show small MAPE. However, P7 and P2 spatial dissimilarity to P3 is high. Therefore, P3 cannot share its model with P2 and P7 as they belong to different clusters.

The same process is applied for computing correlation between temporal parking dissimilarity and parking model performance in Table 7 and between spatiotemporal parking dissimilarity and parking model performance in Table 8.

**Figure 12:**
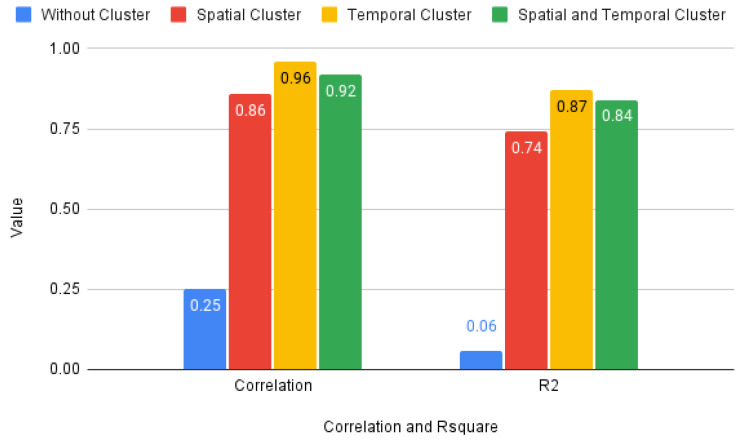
Correlation between dissimilarity matrix and model performance (MAPE).

Based on Table 7 and Table 8, P3, as a parking reference, exhibits smaller dissimilarity to P6 and P16 for temporal clusters, and to P6 for spatiotemporal clusters. Therefore, the model sharing is feasible only for P6 and P16, which belong to the same cluster as P3 for temporal clusters and for P6 in spatiotemporal clusters. However, P2 and P7 cannot share their models with P3 for temporal clusters, and P2, P4, and P16 cannot do so for spatiotemporal clusters. This is because P2 and P7 belong to different temporal clusters, and P2 is in a different spatiotemporal cluster from P3. We have applied the same mechanism to all parking lots in the city and computed the spatial, temporal, and spatiotemporal correlations, with and without clustering, as shown in Figure 12.

Figure 12 illustrates the positive correlation between dissimilarity in terms of spatial, temporal, and both to model performance. Meanwhile, the correlation coefficient calculated based on Equation (6) (see Section 4.3) and coefficient of determination computed using Equation (7) (see Section 4.3) show before clustering was inadequate at only 25% and 6%. However, the correlation coefficient and coefficient of determination significantly improved after clustering.

Temporal clusters have a greater influence on determining dissimilarity between parking lots than spatial factors, as confirmed by higher correlation and $R^{2}$ values for temporal clusters compared to spatial ones. This is also supported by Figure 12, where the correlation coefficient and $R^{2}$ for temporal dissimilarity are closer to the model performance than for spatial dissimilarity.

Therefore, it presents a strong correlation, with values close to 86% for spatial, 96% for temporal, and 92% for both combinations. The strongest values for the coefficient of determination are for temporal clustering (87%), the combination between temporal and spatial (84%), and then spatial (74%). The validation of the result was based on correlation and determination coefficients of the same order, namely temporal, spatiotemporal, and spatial.

Based on these results, we can see the interest of the clustering process for model sharing between parking lots belonging to the same cluster. Parking in the same cluster may indeed be able to reuse a single model that has been trained on similar clusters. Using the clustering mechanism, we have trade-off between training time compared to deploying models for each parking lot in the city and the quality decrease in the clustered model development for several parking lots at once.

A reference model is a selected model in the cluster that has been developed to represent the parking profile at the same cluster. It can be used to create forecasts without any additional tuning. Reference models are typically simple and fast to implement, which means that they can be used to create forecasts quickly and easily. However, because they are not specifically tuned to the particular parking of the cluster, they may not be as accurate as a model that has been specifically developed for that parking. On the other hand, a model that has been specifically tuned for a particular cluster is likely to be more accurate than a reference model. This is because the model has been developed using data that are specific to that parking and tuned to capture the unique characteristics of that parking profile. However, developing and tuning a model for specific parking can be time-consuming and requires a high level of expertise. This means that using a tuned model can take more time and resources than using a reference model. Thanks to our framework, we are now able to exploit a reference model tuned specifically for the parking lots of a given cluster, which allows saving time and resources while maintaining very good accuracy in the forecast.

### 5.4. Model Deployment Cost Analysis

In this section, we analyse the model deployment cost of our approach and compare it with the classical method without any cluster to prove its efficiency. The cost, as we explained before, refers to the total amount of time required for various components involved in the deployment process of the models. The mentioned cost encompasses various factors, such as the time spent on training, validation, and testing the models, the execution time for tuning hyperparameters with different combinations, the time required for updating models, and the deployment time for clustering.

To compute the cost of each mentioned item, we ran our experiment 10 times and then we computed the average in terms of execution time. The obtained results are detailed in Table 9. To run the experiments, we used Google Colab (https://colab.research.google.com/ (accessed on 1 December 2019)), which is a platform that provides free GPU to train deep learning architectures. As a development tool, we used Python and Keras, which is a framework to build deep learning models under the Tensorflow environment.

The values in Table 9 represent the respective time durations (in minutes) for each cost element. The deployment cost without cluster column refers to the cumulative time for the traditional approach without utilising clustering, while the deployment cost with cluster columns indicates the cumulative time for our approach that incorporates different types of clustering (spatiotemporal clusters, spatial clusters, and temporal clusters) to expedite the deployment process. The aim is to compare the time savings achieved by deploying the models using our clustering-based approach in contrast to the classical approach without clustering. In our experiments, we considered seventeen parking lots, which are grouped into six spatiotemporal clusters, three spatial clusters, and two temporal clusters following the clustering process introduced in previous sections.

According to Table 9, we note that deploying the model without clustering incurs a significantly higher deployment cost in terms of time compared to our approach. Specifically, our approach enables deployment that is almost two times faster than the traditional approach when using six spatiotemporal clusters. Moreover, deploying the model with three spatial clusters is three times faster, while using two temporal clusters results in a deployment that is five times faster compared to the traditional approach. Furthermore, the resources consumption is linear to the execution time. The more time consumed to deploy a model, the more resources are required, such as energy, hardware, etc. These findings highlight the efficiency and time and resources saving benefits of our clustering-based approach in reducing the overall deployment cost of the models across multiple parking lots at the city level.

Obviously, reducing the deployment time is useless if the forecasting performance drops significantly. Thus, we also performed a comparison in terms of forecasting error (MAPE) between our approach using clusters and the classical one (without clusters). According to the results of this comparison reported in Table 9, we notice that our approach maintains a comparable performance compared to the classical approach. As a result, we proved that our clustering approach is still efficient in terms of forecasting performance and significantly reduces the time and computing resources needed to deploy the forecasting models.

## 6. Conclusions and Future Work

In this article, we have introduced a novel framework to share parking occupancy forecasting models amongst several parking lots of the same city with similar characteristics. To characterize each parking lot, we have introduced a parking profile to identify their spatial and temporal properties. Our framework exploits these parking profiles to find similarities amongst parking lots and then to group them in different spatial and temporal clusters. Once spatiotemporal clusters are defined, our framework shares a parking forecasting occupancy model for parking lots in the same cluster. The framework is automatically updated when parking lot characteristics change. We have validated our approach by conducting an experimental study on a real-time parking Métropole European Lille (MEL) dataset. We obtained good results, with correlation values of 86% for spatial, 96% for temporal, and 92% for both combinations on sharing parking occupancy models using a clustering approach based on spatiotemporal parking profile similarity at the city level. These findings demonstrate the feasibility of effectively sharing a parking model within a given cluster.

The remaining works related to these approaches are to consider more parameters in spatial profile (i.e, city regulation, tariff, and parking schedule) or temporal profile (i.e, traffic and event). We also observe the possibility to apply transfer learning to focus on improving model performance in the group of similar parking profiles using cluster information. Finally, we can extend our framework for larger instances, such as on-street parking, that have more complex datasets and constraints.

## Figures and Tables

**Table 1:** Hyperparameters tuning search space [8].

Hyperparameter	Values	Combinations
Number of layers	Discrete uniform [3 … 10]	8
Number of neurons per layer	Uniform choice [2, 4, 8, 16, 32, 64, 128]	7
Learning rate	Uniform choice [0.001, 0.003, 0.005,	9
	0.03, 0.05, 0.01, 0.1, 0.3, 0.5]	
Optimizer	Uniform choice [‘ADAM’,	4
	‘ADAGRAD’, ‘RMSPROP’, ‘SGD’]	
Dropout	Uniform [0.1 … 1.0]	10
Recurrent Dropout	Uniform [0.1 … 1.0]	10
Batch Size	Uniform choice [2, 4, 8, 16, 32, 64, 128]	7
Number of epochs	Uniform choice [25, 50, 70, 100]	4
Look-back window	Uniform choice [2, 4, 6, 12, 24, 48, 96, 192]	8
Activation Function	Uniform choice [‘linear’,	6
	‘hyperbolic’, ‘sigmoid’,	
	‘tanh’, ‘SeLu’, ‘ReLu’,]	

**Table 2:** Example of parking profile.

Spatial profile	Max capacity	450
	Longitude	50.6367
	Latitude	3.0743
	Parking nearby	6
	Relevant amenities	Station
	Amenity_types (number)	Station (1)
		Restaurant (6)
Temporal profile	Date and time	2018-12-19 11:16:02, Monday, Weekday
		2018-12-19 11:21:02, Monday, Weekday
		…
		2018-12-25 21:00:10, Sunday, Weekend
		2018-12-25 21:10:02, Sunday, Weekend
	Occupation	(440, 439, …)
	Weather	(0.895, 0.254, …)

**Table 3:** Spatial cluster.

Cluster	1	2	3
Parking	P1, P9, P10, P11, P14, P16	P3, P4, P5, P6, P8, P12, P13, P15, P17	P2, P7
Max Capacity(Avg)	740	480	1900
Latitude (Avg)	50.64563018	50.64945225	50.63175173
Longitude (Avg)	3.092403283	3.0828889	2.99108005
Parking nearby (Avg)	20	3	7
Relevant amenities	Cinema, hotel, stadium, park, market	Restaurant, office, museum, theater, station	Shopping center
Amenity types (Avg number)	22	32	105

**Table 4:** Temporal cluster.

Cluster	Parking	Member
1	P3, P6, P1, P16	4
2	P2, P7, P4, P5, P10, P8, P11, P9, P12, P14, P13	11

**Table 5:** Spatiotemporal clusters.

Type	Temporal Cluster 1	Temporal Cluster 2
Spatial Cluster 1	P1, P16	P14, P11, P10, P9
Spatial Cluster 2	P3, P6	P4, P5, P8, P12, P13, P15, P17
Spatial Cluster 3	-	P2, P7

**Table 6:** Spatial cluster-based model performance evaluation.

Parking	Dissimilarity	Performance	Cluster Label
	P3	P3 (MAPE %)	
P3	0	0.35	Reference
**P12**	**0.591**	**1.53**	**The same spatial**
**P6**	**0.770**	**1.57**	**The same spatial**
P7	1.860	26.44	Different spatial
P2	2.160	45.73	Different spatial

Values in bold prove the efficiency of the spatial clustering approach.

**Table 7:** Temporal cluster-based model performance evaluation.

Parking Lot	Dissimilarity	Performance	Cluster Label
	P3	P3 (MAPE %)	
P3	0	0.35	Reference
**P16**	**0.06**	**4.52**	**The same temporal**
**P6**	**0.006**	**1.57**	**The same temporal**
P2	0.82	26.44	Different temporal
P7	0.18	57.73	Different temporal

Values in bold prove the efficiency of the temporal clustering approach.

**Table 8:** Spatial and temporal cluster-based model performance evaluation.

Parking	Diss S	Diss T	Diss S+T	Performance	Cluster Label
	P3	P3			
P3	0	0	0	0.35	Reference
**P6**	1.08	0.006	**0.77**	**1.57**	**The same spatial and temporal**
P16	1.08	0.06	1.15	4.48	Different spatial the same temporal
P4	0.95	0.03	0.99	4.52	The same spatial different temporal
**P2**	2.16	1.00	**3.16**	**45.73**	**Different spatial and temporal**

Values in bold prove the efficiency of the spatiotemporal clustering approach.

**Table 9:** Comparison of the model deployment cost and forecasting performance of our clustering-based approach with a non-clustering approach.

Items Considered	Deployment Cost without Cluster (Minutes)	Deployment Cost with Cluster (Minutes): Our Approach		
Unit measures	Parking lots	Spatiotemporal	Spatial	Temporal
Number of models	17	6	3	2
Offline Training, Validation and testing	975.2	408.8	204.4	136.3
Hyperparameters tuning	3672.6	2144.7	1072.4	714.9
Updating model (20 epochs)	82.6	38.3	19.1	12.8
Cluster deployment	0	37.2	37.2	37.2
**Total cost (minutes)**	**4730.3**	**2628.9**	**1333.1**	**901.1**
**Total hours**	**78.8**	**43.8**	**22.2**	**15.0**
**Average forecasting error (MAPE)**	**8.2%**	**9.0%**	**8.4%**	**8.7%**

Values in bold highlight the total cost in terms of minutes and hours as well as the average forecasting error.

## Data Availability

The data presented in this study are openly available in https://opendata.lillemetropole.fr/explore/dataset/disponibilite-parkings/information/ (accessed on 1 April 2023).
